# "Contraception" and Consumptives

**Published:** 1913-05-31

**Authors:** Binnie Dunlop

**Affiliations:** Alexandra Court, S.W.


### "Contraception" and Consumptives.

*To the Editor of* The Hospital.

Sir,—I would like to add the following sentences to your interesting article on "Fabian Deliverances on Sanatoria": Few will deny that consumptives frequently transmit to their offspring the susceptibility to tuberculosis. So the more numerous the sanatoria and effective their hygienic-dietetic treatment, the more important is it that discharged patients should refrain from begetting children or further children. In the interests of their families and of the community they should be warned of the risks, and should be given the necessary information about preventive contraceptive methods.

Binnie Dunlop, M.B., Ch.B.

Alexandra Court, S.W., May 24.

